# Viral Cre-LoxP tools aid genome engineering in mammalian cells

**DOI:** 10.1186/s13036-017-0087-y

**Published:** 2017-11-24

**Authors:** Ranjita Sengupta, Amy Mendenhall, Nandita Sarkar, Chandreyee Mukherjee, Amirali Afshari, Joseph Huang, Biao Lu

**Affiliations:** 1 System Biosciences, 2436 Embarcadero Way, Palo Alto, CA 94303 USA; 2 Gilead Sciences Inc., 333 Lakeside Drive, Foster City, CA 94404 USA; 3 Department of Bioengineering, Santa Clara University, 500 El Camino Real, Santa Clara, CA 95053 USA

**Keywords:** Cre-LoxP, Cre recombinase, Lentiviral vector, AAV vector, Genome editing, Talen

## Abstract

**Background:**

Targeted nucleases have transformed genome editing technology, providing more efficient methods to make targeted changes in mammalian genome. In parallel, there is an increasing demand of Cre-LoxP technology for complex genome manipulation such as large deletion, addition, gene fusion and conditional removal of gene sequences at the target site. However, an efficient and easy-to-use Cre-recombinase delivery system remains lacking.

**Results:**

We designed and constructed two sets of expression vectors for Cre-recombinase using two highly efficient viral systems, the integrative lentivirus and non-integrative adeno associated virus. We demonstrate the effectiveness of those methods in Cre-delivery into stably-engineered HEK293 cells harboring LoxP-floxed red fluorescent protein (RFP) and puromycin (Puro) resistant reporters. The delivered Cre recombinase effectively excised the floxed RFP-Puro either directly or conditionally, therefore validating the function of these molecular tools. Given the convenient options of two selections markers, these viral-based systems offer a robust and easy-to-use tool for advanced genome editing, expanding complicated genome engineering to a variety of cell types and conditions.

**Conclusions:**

We have developed and functionally validated two viral-based Cre-recombinase delivery systems for efficient genome manipulation in various mammalian cells. The ease of gene delivery with the built-in reporters and inducible element enables live cell monitoring, drug selection and temporal knockout, broadening applications of genome editing.

**Electronic supplementary material:**

The online version of this article (10.1186/s13036-017-0087-y) contains supplementary material, which is available to authorized users.

## Background

Robust targeted nucleases, including meganuclease, zinc finger nucleases (ZFN), transcription activator-like effector nucleases (TALEN) and clustered regularly interspaced short palindromic repeat-associated nuclease Cas9 (CRISPR-Cas9), are providing modern tools for genome engineering [1–4]. Remarkably, targeted nucleases not only can be programmed to bind specific gene loci within a complex mammalian genome, but they can also generate bi-allelic changes via their nuclease activities [5, 6]. This new capability allows efficient generation of knockout animals and works directly on a variety of species including human cells and disease cell lines [7–10]. In fact, in combination with homologous recombination, targeted nucleases have been used for genome editing to elucidate molecular mechanisms of many biological and disease processes using cultured cell models [5, 8]. By themselves alone, however, targeted nucleases can only produce double stranded DNA breaks, which in turn stimulate error-prone non-homologous end- joining (NHEJ), resulting in small sequence changes, termed indels, near the binding sites [11, 12]. In combination with Cre-LoxP technology, the targeted nucleases could potentially be used in advanced and complicated genome editing to generate large and precise changes such as inframe fusion or single-nucleotide corrections [10, 13–19].

Cre-LoxP is a site-specific recombinase technology, used to carry out deletions, insertions, and inversions at specific sites in transgenic mice [20–23]. Cre-LoxP consists of a nuclease, the Cre Recombinase derived from P1 bacteriophage, which recognizes and catalyzes recombination between two LoxP recognition sites [20, 24]. Each LoxP site consists of a 34 bp consensus sequence with an 8 bp core spacer sequence flanked on either side by a 13 bp palindromic sequence [25, 26]. When a DNA element is flanked by two LoxP sites, the Cre recombinase recognizes the lox P sites and cuts it. The DNA element that is present between the two sites gets removed and the flanking sites get fused together. The orientation and location of the LoxP sites determine whether Cre recombination induces a deletion, inversion, or chromosomal translocation [21, 27]. These predictable changes can be utilized to generate conditional knockouts as well as fusion reporters to study gene function and regulation. Traditionally, the Cre-LoxP system is a mainstay method for generating conditional knockouts in mice [27]. Through homologous recombination, LoxP sequences are first introduced into the animal genome flanking the gene of interest. Subsequent selection and production of homozygous off-spring allow conditional knockouts via the expression of Cre-recombinase. Although the Cre-LoxP system is used routinely in transgenic animals, its application in cultured mammalian cells coupled with targeted nucleases has grown rapidly in recent years [10, 17, 28, 29]. This is because Cre and targeted nucleases (ZFN, TALEN, and Cas9) have different advantages and disadvantages depending on applications [19, 26]. Cre is restricted in editing LoxP-flanked sequences, but its predictability, specificity and robustness in removing or reversing the floxed sequences could not be easily achieved by the targeted nucleases due to a lack of both specificity and precision of these targeted nucleases [3]. Therefore, adding of Cre will allow additional genome editing ability and enable complicated and advanced genome manipulation especially when floxed sequences are present [19]. However, a lack of robust and easy-to-use Cre-delivery system imposes a great hindrance to its implementations. However, a lack of robust and easy-to-use Cre-delivery system imposes a great hindrance to its implementations.

To address this issue, we combined the Cre-LoxP gene editing technology with two very powerful viral gene delivery methods, the lentiviral and adeno associated viral (AAV) technologies. Both these viral systems have been demonstrated to deliver transgenes into a vast variety of mammalian cell types with distinctive characteristics [30–32]. The integrative nature makes lentiviral delivery highly effective while non-integrative AAV provides a safer means for potential therapeutic applications [33–35]. To take advantage of both systems, we designed and constructed two sets of Cre-recombinase expression vectors. To enable live cell monitoring and facilitate engineered cell enrichment, we further designed both vector sets with either green fluorescent protein (GFP) and/or Puromycin-resistant gene (Puro).

Here, we report the development and potential use of two novel viral-based Cre-delivery systems in cultured mammalian cells. We validated the ability of these two Cre-delivery systems in the removal of Floxed sequences using our previously established TALEN-edited human HEK293 cells [28]. Our study demonstrates that the floxed reporter genes introduced by targeted nucleases and the homologous donor can be successfully removed by viral delivery of Cre-recombinase in HEK293 cells. This new resource will provide researchers a robust and easy-to-use method to conduct more complicated and advanced genome engineering.

## Results and discussions

### Design and construction of Cre-delivery vectors

In order to establish a robust and easy-to-use Cre-delivery system for gene editing, we used three state-of-the-art technologies in this study: i) the precise cutting ability of targeted nucleases (TALEN) for double-stranded DNA [28]; ii) the site-specific recombinase of Cre-LoxP, and iii) two powerful viral gene delivery systems, lentivirus and AAV delivery.

**Fig. 1:**
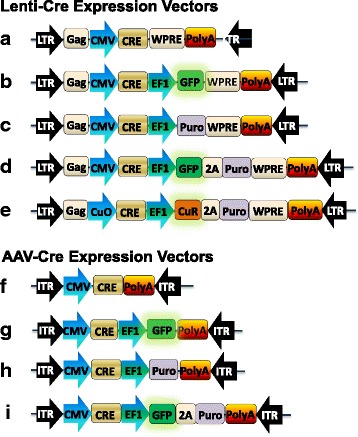
Schematic illustration of design and construction of recombinant AAV and lentiviral vectors for Cre expression. Features of lentiviral vectors, depicting the humanized Cre gene under the control of the CMV promoter without a selection marker (**a**) or in a combination with optional selection markers, including GFP (**b**), Puro (**c**), GFP plus Puro (**d**). The Cumate-inducible expression of CRE using an all-in-one lentiviral vector (**e**). Similarly, features of the AAV vectors, depicting the humanized Cre gene under the control of the CMV promoter alone (**f**) for with optional selection markers GFP (**g**), Puro (**h**), or GFP plus Puro (**i**)

First, we designed and constructed five lentiviral vectors for expressing Cre recombinase (Cre) with single or dual promoters (Fig. 1 a-e). One vector contains a CMV promoter driving Cre (Cre sequences in Additional file 1) expression and a WPRE at the 3’end of the construct (Fig. 1a). Three vectors are dual promoter constructs with CMV driving Cre expression and EF1α driving GFP and/or puro with WPRE at the 3′ end of the marker gene (Fig. 1b-d). The fifth construct is an inducible vector where Cre was cloned into the All-in-one Cumate switch inducible lentivector (Fig. 1e). In this Cumate inducible vector, Cre is driven by the Cumate Operator (CuO) which is turned on when Cumate is added (System Bioscience Inc. (SBI), Palo Alto, CA), while EF1 drives expression of the CymR repressor and Puromycin separated by a ribosomal skip site T2A (Fig. 1e). For lentiviral vectors, the WPRE mRNA stabilization sequence was placed 5′ to the poly-A signal.

Using a similar strategy, the AAV vectors for expressing Cre are configured from 5′ to 3′ as follows: 5′ inverted terminal repeat (ITR), a constitutive cytomegalovirus promoter (CMV), Cre, a poly A signal (poly-A), and 3’ITR (Fig. 1f-i). The marker genes GFP and/or Puro for live cell monitoring and/or drug selection respectively are included under the constitutive EF1α promoter (Fig. 1f-i). T2A is placed between GFP and puro in constructs containing both markers.

The authenticity and accuracy of all these expression vectors were verified by full sequencing prior to functional testing.

### System setup for functional testing of Cre-delivery vectors

**Fig. 2:**
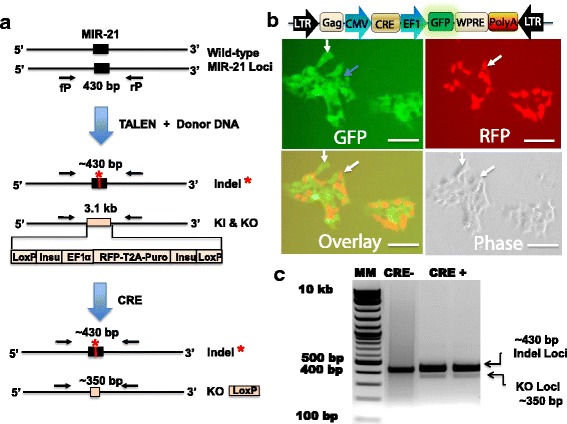
Strategy for establishing TALEN-engineered HEK293 reporter for testing Cre function. **a** The schematic flowchart shows the unmodified HEK293 cells harboring the wild-type MIR-21 gene at both loci (*upper panel*). Introduction of TALEN and HR donor DNA results in bi-allelic ablation of MIR-21 [28]. Genome sequence confirmed an indel of 5 bp at one allele and a knockin (KI) and knockout (KO) event at the other allele via homologous recombination (*middle panel*). These TALEN-engineered HEK293 (HEK293-TE) cells may serve as a reporter cell line for functional test of the CRE activities (*lower panel*). **b** The lenti-Cre-GFP vector is introduced into the HEK293-TE cells by transfection. Green fluorescence shows the expression of Cre-GFP (*upper left)* and red fluorescence shows the expression of floxed-RFP-Puro *(upper right). *The overlay shows that the Cre-GFP expressing cells do not overlap with the Floxed-RFP-Puro cells*(lower left*). **c** Genotyping of the HEK293-TE cells after Cre-GFP introduction. The upper ~430 bp is the indel allele and lower ~350 bp is corresponding to the smaller PCR product resulting from the removal of the floxed reporter cassette. The Foxed reporter cassette (3.1 kb) allele could not be amplified under our experimental conditions. White arrows show GFP and RFP positivity are mutually exclusive. Scale bar 50 μm

Targeted nucleases have been successfully used to knock in or knock out genes for cancer research, developmental studies and gene therapy, and can produce bi-allelic changes via their robust nuclease activities [2, 5, 8]. We previously accomplished bi-allelic gene ablation of MIR-21 gene using a TALEN pair and a homologous recombination donor in HEK293 cells (Fig. 2a) [28]. Using this established TALEN-engineered HEK293 cells (HEK293-TE), in this study we developed a strategy for monitoring Cre activities for our newly designed and constructed Cre expression vectors. This reporting strategy is critical and highly relevant to our study, as it clearly demonstrates the power of combining targeted nucleases technology with Cre-LoxP and viral delivery system to fulfill advanced genome editing.

To test the feasibility of this approach, we transfected red fluorescent HEK293-TE cells with a lentivector expressing both Cre and GFP (Fig. 2b **, upper right**). Genome typing and sequence analysis show an indel on one allele and a knockin and knockout change on the other allele in one HEK293-E clone (Fig. 2a **upper and middle panels**; Fig. c). By visual observation, a fraction of cells showed green fluorescence on Day 1 following the transfection, indicating a fast onset of transgene (Cre) expression using a transient transfection protocol. On Day 2, the green fluorescence became stronger while the red fluorescence became weaker, suggesting the removal of floxed RFP-Puro from the engineered cells. On Day 3, cells expressing Cre-GFP become evident and distinctive when comparing fluorescence signals of RFP, as recorded in Fig. 2b **(upper left**). The red and green fluorescence do not overlap in the same field, indicating that these cells still express either the floxed-RFP-Puro or Cre-GFP (Fig. 2b **, lower panels**). We contribute the mutual exclusive phenomena to the successful delivery and expression of robust Cre gene and the subsequent removal of floxed-RFP-Puro sequences. This transfection data shows the effectiveness of Cre-LoxP technology.

Next, we verified the removal of floxed RFP-Puro in HEK293-TE reporter cells by genome typing analysis. In both TALEN- and Cre-engineered cells, the indel loci (~430 bp) should be present at all times, representing the PCR product of NHEJ-modified allele. Under our PCR condition, the knockin allele is intended not to be amplified by limiting the extension time of PCR. Thus, while TALEN-engineered cells will show one PCR product, the Cre-engineered cells is expected to show two PCR products, including the indel (~430 bp) and successful removal of knockin floxed RFP-Puro cassette (~350 bp). As predicted, the knockout loci (KO, ~350 bp) appears only after the introduction of Cre into the cells (CRE+ lanes), and represents the removed region of the floxed-RFP-Puro cassette (Fig. 2c). These results confirm that Cre is editing out floxed sequences in engineered cells. Additionally, this fluorescent toggling provides an easy way to real time monitor the activities of Cre in live mammalian cells. Therefore, we used the fluorescence features of these reporter cells to conduct the rest of our studies with microscope.

### Lenti-Cre efficiently removes floxed RFP-puro in HEK293-TE reporter cells

**Fig. 3:**
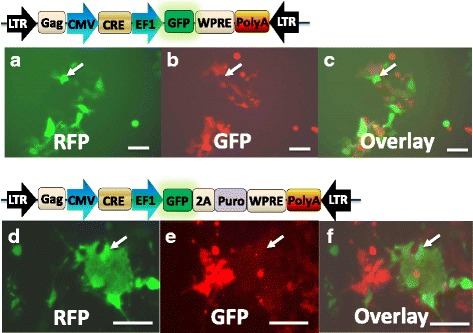
Efficient removal of floxed-reporter genes with lentiviral transduction protocol. Functional validation of Cre-expression vectors using viral transduction. HEK293-TE cells at 20~40% confluency were transduced (0.5 MOI)with either lenti-Cre-GFP (**a-c**) or lenti-Cre-GFP-Puro (**d-e**) for 5 days and images were taken at 20× magnification. Green fluorescence indicates the expression of Cre-GFP or Cre-GFP-Puro (**a, d**). Red fluorescence indicates the expression of floxed-RFP-puro (**b, e**). Overlay of either A and B, or D and E, respectively, shows that the Cre-GFP expressing cells do not overlap with the floxed-RFP (**c**) or floxed-RFP-puro (**f**) cells. White arrows show GFP and RFP positivity are mutually exclusive. Scale bar 50 μm

Previously we and others demonstrated that the vesicular stomach virus envelope glycoprotein (VSVG)-pseudotyped lentiviruses have a super high infection rate of ~100% [36, 37]. In this study we chose VSVG-pseudotyping protocol for lenti-Cre virus production, and examined whether VSVG-pseudotyped lenti-Cre-GFP could deliver, express and subsequently edit the knockin floxed-RFP-Puro in reporter cells. We transduced HEK293-TE reporter cells with a low MOI of 0.5 to test the efficiency and robustness of lenti-system. As predicted, we initially observed a weak transgene expression (GFP) at Day 2 after viral transduction. The GFP signal became stronger and evident on Day 3, indicating a relative slower process of transgene expression compared to the transfection protocol described above. In the meantime, the RFP signal became weaker in the GFP positive cells. On days 5–7, fluorescent signals became strong and distinctive for either GFP or RFP (Fig. 3a-c **)**. The mutual exclusiveness of GFP and RFP fluorescence showing in these lentiviral infection data demonstrates the robust and efficient gene editing of this lenti-Cre delivery system. Under the same experimental condition, another lenti-Cre-GFP-Puro vector demonstrates the equivalent activity in the removal of floxed RFP-Puro in a similar fashion (Fig. 3d-f). These results lead to two important conclusions: first, lenti-delivery to recipient cells is robust; second, the onset of Cre-editing may be delayed as compared to that of transfection protocol. The delay is most likely the time it takes for the lenti-Cre to integrate into the host genome. In transfection, however, the expression of Cre starts immediately because they are independent of integration. In summary, transfection protocol has a rapid onset of transgene expression with moderate transfection rate, which may be suitable for easy-to-transfect cell types, while transduction protocol has a delayed onset of transgene expression with a high transduction rate, which may be applicable to more difficult-to-transfect cell types. In addition, to take advantage of optional markers, both our lenti-Cre protocols are easily amenable to fluorescence activated cell sorter (FACS) or simple drug selection protocol. Together, our lenti-Cre system provides a robust and flexible solution to advanced genome editing in mammalian cells.

### AAV-Cre removes floxed RFP using both transfection and transducing protocols in HEK293-TE reporter cells

**Fig. 4:**
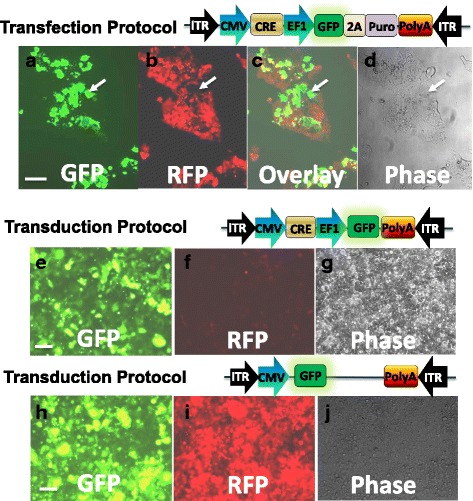
Functional Validation of AAV-based Cre in engineered HEK293 cells. HEK293-TE cells were transfected with the constructs expressing Cre and GFP (**a-d**). Images were taken at 20× magnifications, 3 days after transduction. Green fluorescence depicts cells expressing Cre-GFP-puro (**a**). Red fluorescence depicts cells expressing floxed-RFP-puro (**b**). Overlay of the left and middle images, respectively shows that cells either express RFP or GFP (**c**), and the phase contract of overlay (**d**). Alternatively, cells at 20~30% confluency were transduced with the AAV (2.0 MOI) expressing Cre-GFP (**e** to **g**) or the control GFP viruses (**h** to **j**). Images were taken at 20× magnification, 10 days after transduction Green fluorescence indicates the expression of Cre-GFP (**e**) or control GFP (**h**). Diminished red fluorescence indicates the expression of floxed-RFP-puro (**f**). In contrast, red fluorescence signal remains strong in GFP non-editing control (**i**). Phase contrast shows the TALEN-Cre sequential edited HEK293 cells (**g**) vs. control cells (**j**). White arrows show that GFP and RFP positivity are mutually exclusive. Scale bar 50 μm

In contrast to integrated lentivirus, AAV allows delivery of transgenes into cells without integrating into the host cell genome. We next studied genome-editing activities of AAV-Cre in HEK293-TE reporter cells. First, we tested whether AAV vectors can be used by simple transfection protocol. As shown in Fig. 4, transfection with AAV-Cre-GFP-Puro resulted in the appearance of GFP in a fraction of cells (Fig. 4a), parallel with the disappearance of RFP in the same cell population (Fig. 4b-d), suggesting the successful removal of floxed RFP-Puro from the engineered cells by introduction of Cre gene into the cells.

The success in our AAV-Cre transfection protocol encouraged us to further examine the performance of these vectors on HEK293-TE cells by transduction protocol. Despite the use of AAV technology in vivo, the infection rate of AAV is not as efficient as lenti virus and is relatively low in in vitro cell culture conditions. However, since AAV has low toxicity, we transduced reporter cells with high dose of viruses (2 MOI; using either Cre-GFP editing or GFP non-editing control viruses). Consistent with a weaker infectivity under cell culture conditions, the expression of transgene GFP appears on Day 3 and became stronger on Days 4~5 (Fig. 4, e-j**)**. The disappearance of RFP however, occurred on Days 6~7 after transduction (data not shown). On Day 10, almost all cells become GFP-positive and RFP-negative in the Cre group (Fig. 4e-g **)**, suggesting the successful removal of floxed RFP-Puro from HEK293-TE cells transduced with Cre-GFP editing virus. In contrast, cells become GFP-positive (Fig. 4h) but remain RFP-positive (Fig. j) in GFP non-editing control group. Together, we validated the AAV-Cre delivery system using both easy-to-perform transfection and viral transduction protocols. The non-integrative nature of AAV makes it particularly useful for clinical applications.

### Lenti- and AAV-based systems provide a comprehensive solution to advanced genome editing in various mammalian cells

In this study, we explored the combination of three advanced technologies: targeted nucleases, Cre-LoxP and lentiviral/AVV delivery systems, and successfully established a robust and easy-to-use Cre-delivery system for gene editing in mammalian cells. Targeted nucleases have been the core technologies to knockin or knockout genes for developmental studies, cancer research and gene therapy [10, 13]. Genome modifications producing bi-allelic changes can be made through targeted genome cleavage by engineered, sequence-specific nucleases such as TALENs [2]. Cre-LoxP is another genome editing mechanism with unique advantages. First, it is a simple two-factor system requiring only the Cre recombinase enzyme and two LoxP sites. Second, it can be used in any cellular environment including mammalian cells, yeast cells, and has applications both in vitro and in vivo. Third, it also can be made conditionally by incorporating to an inducible vector such as Cumate-operator system. Finally, the Cre-LoxP system can work in quick, effective and precise manners by transfection protocols in easy-to-transfect cell line such as HEK293 cells.

Previously we had developed a system for delivering various genes into cells using either lentiviral or AAV particles [36, 38]. We initially selected the lentiviral system because of its favorable features: (1) Lentiviral transduction is an efficient way to create a stable cell line expressing Cre in almost any mammalian cell type including stem cells and non-dividing cells. Results from these studies indicate that the onset of expression and removal of floxed RFP in lentiviral transduced cells is faster than in AAV (5 vs. 7–10 days). (2) Lentiviral system can be customized with a Cumate-inducible promoter driving expression of Cre, enabling scientists to follow biological changes induced by Cre in real time, thus expanding its application (Additional file 2: Figure S1). (3) There is a newly available integrase-defective lentiviral packaging system, which enables transduction of larger lentiviral constructs without undesired integration of the lenti construct into the genome [36]. In contrast, AAV vectors have a smaller gene packaging size of ~ 3.7 kb, which make them unsuitable for building all-in-one inducible Cre (~7.0 kb in size). However, AAV system has its own advantages: (1) they can easily deliver Cre into their target cells without integration into the host cell genome, thus enabling experiments targeted for potential clinical applications such as gene therapy or therapeutic purposes. To make use of the packaging capacity of lentivector, we have built a full suite of expression vectors with/without selection makers GFP and/or Puro (Additional file 3: Figure S2A-C), thus providing options that can meet different research needs. Furthermore, the broad tropism (Additional file 3: Figure S2D-G) coupled with titratable feature of AAV-based vectors (Additional file 3: Figure S2h-k) make them desirable for delicate applications with less genome toxicity [39].

## Conclusions

We developed and functionally validated two effective viral Cre-delivery systems with two different flavors (Lenti and AAV) for advanced genome-editing in mammalian cells. These novel methods combine sequence-specific editing ability of Cre with targeted nucleases, which enables complicated genome manipulation in a vast variety of cell types. The built-in reporters and inducible elements designed in the lenti and AAV vectors enable live cell monitoring, drug selection and temporal knockout. Although both CRISPR-Cas9 and TALEN technologies are getting more popular, their ability in editing floxed sequences is limited and thus requires Cre to carry out specific action under these situations. The precise sequence editing ability of Cre coupled with efficient delivery to various mammalian cell types make these systems very attractive, rendering broad applications and generating sophisticated but biologically relevant results.

## Materials and methods

### Design and construction of lentiviral and AAV vectors for Cre-recombinase

Five lentiviral vectors expressing Cre recombinase (Cre) were designed and constructed with single or dual promoters **(**Fig. 1A-E **)**. Using similar strategy, five AAV vectors for expressing Cre recombinase were designed and constructed (Fig. 1 f-i). The construction of all vectors was performed by PCR amplification of fragments and were joined together using a seamless cloning kit (SBI, Cat# MC100A-1) as reported previously [40]. All final constructs were verified by full sequencing.

### Cell culture, transfection, and Cumate induction

Human embryonic kidney cells (HEK293) were cultured and maintained in high glucose Dulbecco’s Minimal Essential Medium (DMEM) supplemented with 10% FBS, 2 mM GlutaMax, 100 U/ml penicillin and 100 U/ml streptomycin. The engineered cells expressing RFP and puromycin resistance were maintained in the presence of 5 μg/ mL puromycin but switched to normal complete medium at least 2 passages before experimentation. Two different HEK293 cells were used in this study: TALEN-engineered HEK293 expressing RFP (HEK-293TE) [28] and 293TN cells (SBI, Cat# LV900A-1) for viral packaging and AAV titration.

All transfections were performed in 6-well plates. Cells were plated at a density of 2 × 10^5^ cells per well on the day before transfection. Cells were at 30 ~ 50% confluency the next day and transfected with plasmid (2 μg), using Purefection transfection reagent (SBI, Cat#LV850A-1) according to manufacturer’s instructions. For virus transduction, cells were plated at a lower density of 0.5× 10^5^ cells per well on the day before transduction. Cells expressing inducible Cre constructs were treated with 5× Cumate for induction experiments (SBI, Cat#QM100A-1).

### Lentiviral packaging and titration

Lentiviral vector constructs were packaged into lentivirus using pPACKH1 (SBI, Cat# LV500A-1) as previously reported [36, 41]. 293TN cells were transfected with lenti-vector and pPACK-H1, lentivirus packaging mix. 48 and 72 h post transfection, cell culture media containing Lentivirus was collected at and concentrated using PEG-it (SBI, Cat# LV810A-1). The viral pellet was suspended in sterile PBS and tittered using the Global Ultra-Rapid Tattering kit (SBI, Cat# LV961A-1). These concentrated lentiviruses were used to infect the 293TN cells.

### AAV production and titration

293TN cells were plated in a 10 cm dishes to reach a confluency of 70 ~ 80% in DMEM growth media with 10% FBS and 5% Glutamax overnight. Cells were transfected the next day with 12 μg each of the following: AAV vector, pAAV-RC (Cell Biolabs, Inc., San Diego, Cat# VPK-422) and AAV helper plasmids (Cell Biolabs, Inc., Part No.340202) at a 1:1:1 ratio, for a total of 36 μg of plasmid DNA., The media was changed to complete DMEM with Penn/Strep 18 to 24 h after transfection. 72 h post transfection, the media was collected and briefly spun down at 3000 rpm for 15 min to get rid of cell debris. AAVanced™ Concentration Reagent (SBI, Cat# AAV100A-1) was added according to the manufacturer’s instructions. The viral pellet was re-suspended in a small volume of sterile PBS. This concentrated AAV was used to infect the HEK293-TE cells. AAV-DJ helper free packaging system was used unless otherwise indicated.

Both green cell fluorescent assays and PCR were used to determine the multiple of infection (MOI) as reported [38]. For the green cell fluorescent assays, 293TN cells grown on 12-well plates were infected with serial dilutions of CMV-GFP-2A-Luciferase positive control virus. 72 h later, cells infected with GFP-positive virus were visually scored using a fluorescence microscope, and the viral MOI was determined by GFP positive cells. The MOI of AAV-reporter was estimated by the relative copy number of recombinant virus versus the positive control viruses as reported [42, 43].

### Genotyping to confirm the removal of floxed RFP-puro reporter genes from genome

To confirm the removal of floxed RFP-puro (3.1 kb), the MIR-21 gene loci were PCR-amplified from genomic DNA of Cre-transduced cells and the control cells using the EZ Genotyping kit (Cat# GE200A-1,SBI) according to the manufacturer’s instructions. To determine genome types, a pair of primers (forward primer, fP: 5′-TGGGGTTCGATCTTAACAGG-3′ and reverse primer, rP: 5′-TTTCAAAACCCACAATGCAG-3′) was used to perform PCR on the MIR-21 loci. PCR products with sizes of 430 bp for the indel allele or 350 bp for the floxed-RFP-puro cassette-removed allele were selectively amplified under the condition: 94 °C, 30 s; 60 °C, 30 s; and 72 °C, 10 s, for a total of 30 cycles. The products were then subject to 2% agarose gel electrophoresis in 1 × TAE buffer.

### Microscopy and live cell monitoring

All microscopy was performed on live cells using a LEICA DMI3000B fluorescent microscope. Data collection and processing were performed with LAS 3.8 software. The same field was subject to imaging under different conditions such as phase contrast, GFP and/or RFP. Imaging was further processed and overlaid using Adobe Photoshop CS or MS PowerPoint program to illustrate the relationships of GFP and/or RFP positivity.

## Additional files

Additional file 1: Coding and amino-acid sequences of Cre recombinase. (PDF 40 kb)

Additional file 2: Figure S1. The conditional removal of floxed RFP from HEK293 genome by Cumate-induction. (PDF 412 kb)

Additional file 3: Figure S2. Features of AAV-based Cre-delivery vectors. (PDF 367 kb)

## Abbreviations

CMV: cytomegalovirus promoter
EF1α: Elongation Factor 1-alpha promoter
FBS: fetal bovine serum
GFP: green fluorescent protein
HEK293: human embryonic kidney cell line
HEK293-TE: TALEN-engineered HEK293 cell
ITR: inverted terminal repeats
LTR: long terminal repeats
mCMV: minimal CMV promoter
Puro: puromycin-resistant gene
RFP: red fluorescent protein
T2A: self-cleaving 2A peptide sequence
TALEN: Transcription activator-like effector nuclease
TF: transcription factor
