# Supplementary material for: Viral Cre-LoxP tools aid genome engineering in mammalian cells
Source: J Biol Eng. 2017 Nov 24;11:45. doi: 10.1186/s13036-017-0087-y (PMC5702101; doi:10.1186/s13036-017-0087-y)
Supplement: Supplementary file 1 — Coding and amino-acid sequences of Cre recombinase. (PDF 40 kb) [file 13036_2017_87_MOESM1_ESM.pdf]

>Cre coding sequences

gccaccATGtccaatttacttaccgtacaccaaaatttgcctgcattaccggtcgatgcaacgagtgatgaggttcgcaagaacctgatggacatgtt
cagggatcgccaggcgttttctgagcatacctggaaaatgcttctgtccgtttgccggtcgtgggcggcatggtgcaagttgaataaccggaaatggtt
tcccgcagaacctgaagatgttcgcgattatcttctatatcttcaggcgcgcggtctggcagtaaaaactatccagcaacatttgggccagctaaacat
gcttcatcgtcggtccgggctgccacgaccaagtgacagcaatgctgtttcactggttatgcggcggatccgaaaagaaaacgttgatgccggtgaac
gtgcaaaacaggctctagcgttcgaacgcactgatttcgaccaggttcgttcactcatggaaaatagcgatcgctgccaggatatacgtaatctggcat
ttctggggattgcttataacaccctgttacgtatagccgaaattgccaggatcagggttaaagatatctcacgtactgacggtgggagaatgttaatcc
atattggcagaacgaaaacgctggttagcaccgcaggtgtagagaaggcacttagcctgggggtaactaaactggtcgagcgatggatttccgtctct
ggtgtagctgatgatccgaataactacctgttttgccgggtcagaaaaaatggtgttgccgcgccatctgccaccagccagctatcaactcgcgccctg
gaagggatttttgaagcaactcatcgattgatttacggcgctaaggatgactctggtcagagatacctggcctggtctggacacagtgcccgtgtcgga
gccgcgcgagatatggcccgcgctggagtttcaataccggagatcatgcaagctggtggctggaccaatgtaaatattgtcatgaactatatccgtaa
cctggatagtgaaacaggggcaatggtgcgcctgctggaagatggcgatTAG

>Cre recombinase protein sequences contain 343 amino-acids

MSNLLTVHQNLPALPVDATSDEVRKNLMDMFRDRQAFSEHTWKMLLSVCRSWAAWCKLNNRKWFPAEPEDVRDY
LLYLQARGLAVKTIQQHLGQLNMLHRRSGLPRPSDSNAVSLVMRRIRKENVDAGERAKQALAFERTDFDQVRSLMENS
DRCQDIRNLAFLGIAYNTLLRIAEIARIRVKDISRTDGGRMLIHIGRTKTLVSTAGVEKALSLGVTKLVERWISVSGVADDP
NNYLFCRVRKNGVAAPSATSQLSTRALEGIFEATHRLIYGAKDDSGQRYLAWSGHSARVGAARDMARAGVSIPEIMQA
GGWTNVNIVMNYIRNLDSETGAMVRLLEDGD*

Note: Cre coding sequences (cds) are indicated by capitalized start (ATG) and stop (TAG) codons. The cds are in put in a context of Kozack consensus sequences, which are underlined.
