# Supplementary material for: Viral Cre-LoxP tools aid genome engineering in mammalian cells
Source: J Biol Eng. 2017 Nov 24;11:45. doi: 10.1186/s13036-017-0087-y (PMC5702101; doi:10.1186/s13036-017-0087-y)
Supplement: Supplementary file 2 — The conditional removal of floxed RFP from HEK293 genome by Cumate-induction. (PDF 412 kb) [file 13036_2017_87_MOESM2_ESM.pdf]

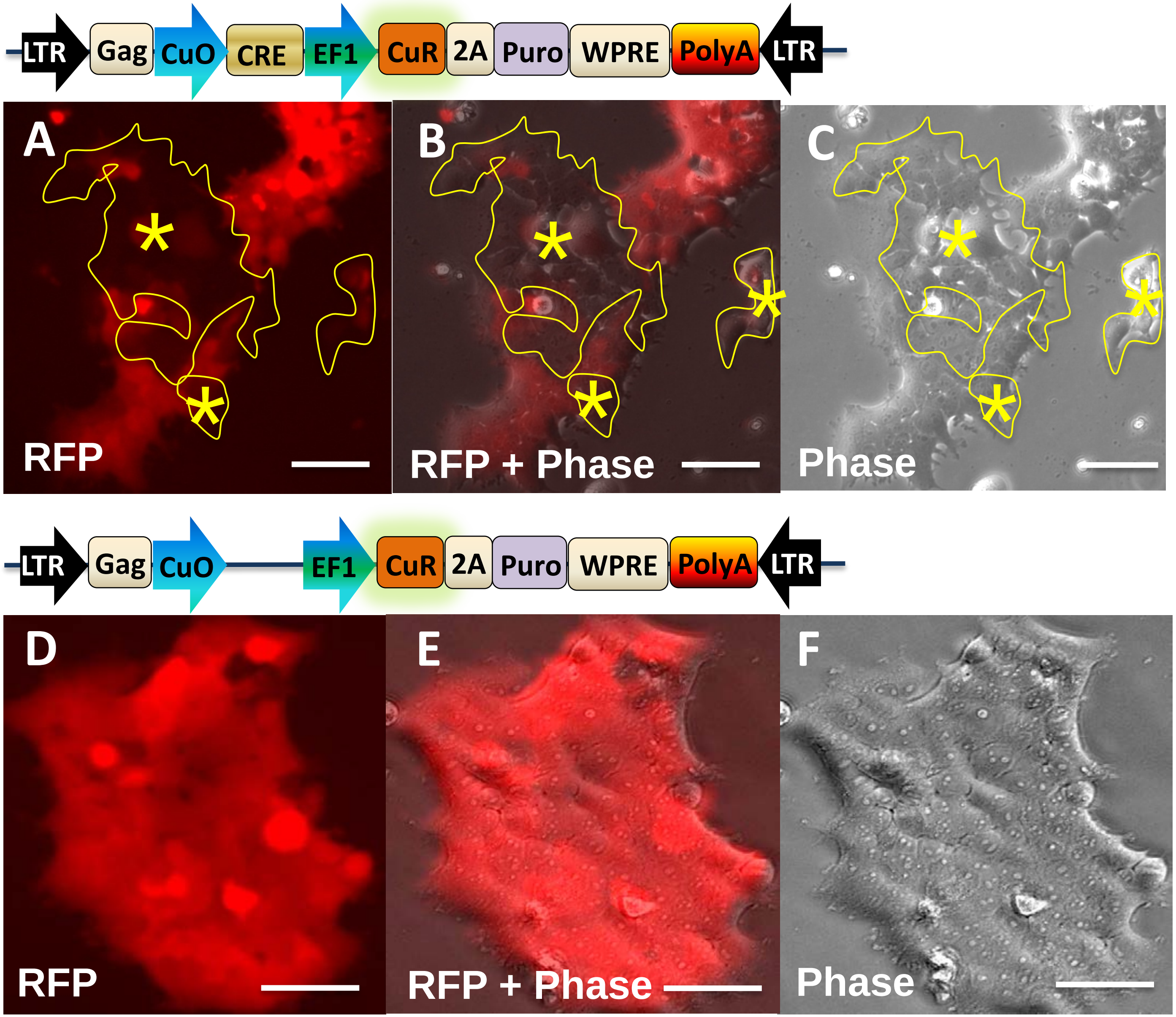

Supplementary Figure 1. Conditional removal of Floxed RFP from engineered HEK293 genome by cumate induction. Engineered HEK293 cells were transfected with cumate inducible Cre-lentivector (**A-C**) or control plasmid DNA without Cre for 24 hours (**D-F**). Cumate was added to induced the Cre expression. Images were taken for live cells in culture at 5 days following induction. The disappearance of RFP in cells (outlined in yellow and marked with *), indicating the removal of floxed RFP in the transfected cells (**A-C**). Under the same experimental condition, the control panels show no significant disappearance of RFP in imaged cells (**D-F**).
