# Supplementary material for: Viral Cre-LoxP tools aid genome engineering in mammalian cells
Source: J Biol Eng. 2017 Nov 24;11:45. doi: 10.1186/s13036-017-0087-y (PMC5702101; doi:10.1186/s13036-017-0087-y)
Supplement: Supplementary file 3 — Features of AAV-based Cre-delivery vectors. (PDF 367 kb) [file 13036_2017_87_MOESM3_ESM.pdf]

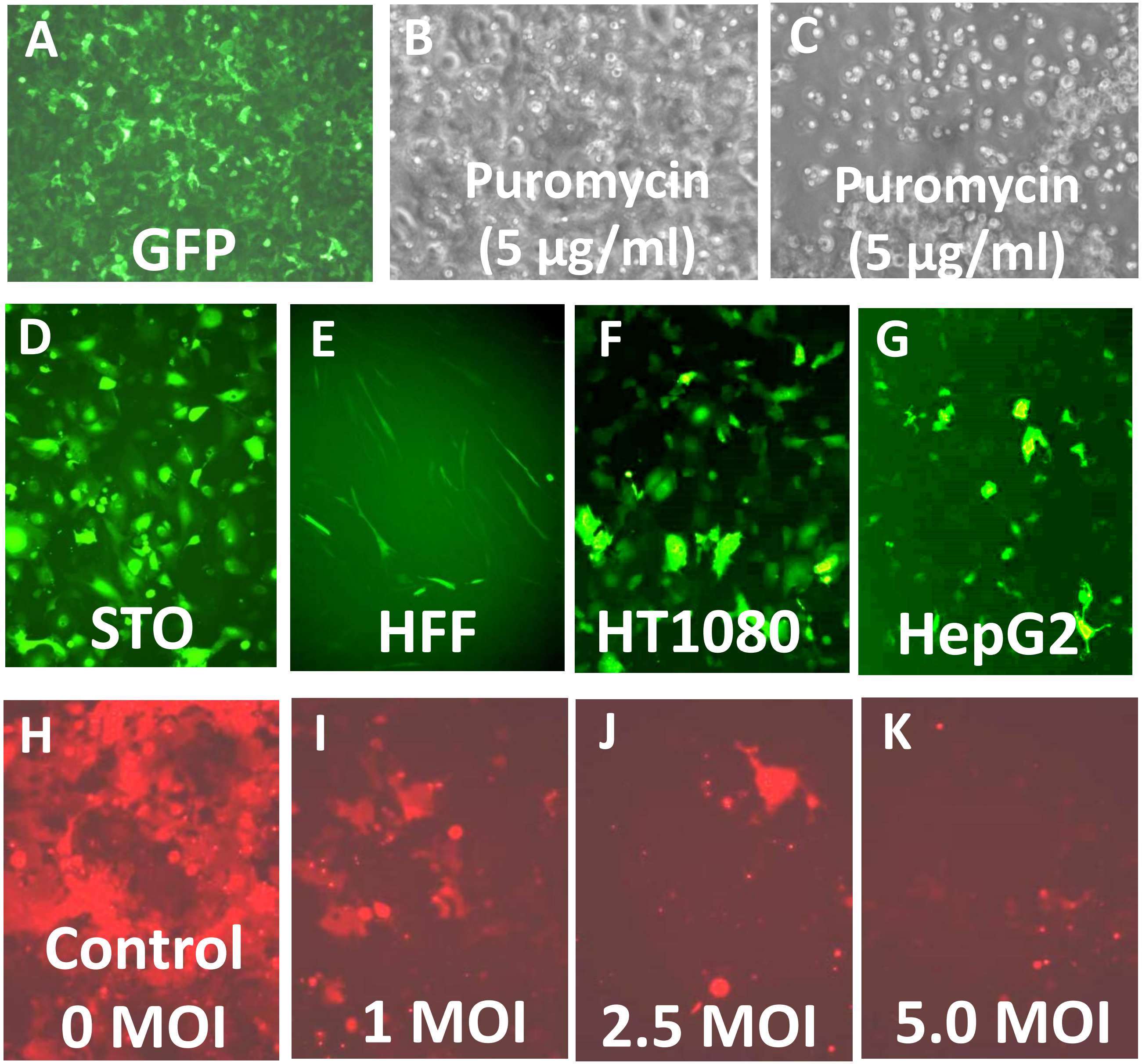

Supplementary Figure 2. Features of AAV-based Cre-delivery vectors. Optional markers provide a visual means for monitoring of transduced HEK293 cells (**A**, GFP) or renders drug-resistance for enrichment (**B**, Purocycin resistance). Non-transduced control cells are Puro-sensitive parental HEK293 and cells die in the presence of the drug for 24 hours (**C**). Transduction of several cell types using the AAV Cre-GFP viruses demonstrates broad tropism (STO, mouse fibroblasts; HFF, human foreskin fibroblast; HT1080, fibrosarcoma cell line; HepG2, human liver carcinoma cell line) (**D-G)**. The complete removal of Floxed GFP from the HEK293 genome by simply increasing the amounts of viral particles, demonstrating titratability (**H-K)**.
